# Experimental Values for the Elastic Constants of a Particulate-Filled Glassy Polymer

**DOI:** 10.6028/jres.080A.008

**Published:** 1976-02-01

**Authors:** Jack C. Smith

**Affiliations:** Institute for Materials Research, National Bureau of Standards, Washington, D.C. 20234

**Keywords:** Composite materials, elastic constants, filled polymers, mechanical properties, particulate composites, Poisson’s ratio, Young’s modulus

## Abstract

Young’s modulus and Poisson’s ratio have been measured simultaneously on a series of particulate composites containing volume fractions of filler up to 0.50. The composites consisted of small glass spheres imbedded in a rigid epoxy polymer matrix. The measured values were compared with theoretical values calculated from current theories. A recently generalized and simplified version of van der Poel’s theory provided the best agreement. It predicted values of Young’s modulus for composites with filler volume fractions up to 0.35. Measured values of Poisson’s ratio exhibited scattering, but were consistent with values calculated from van der Poel’s theory.

## 1. Introduction

It has been known for some time that the addition of a particulate filler to a polymeric material can greatly affect the elastic properties of the resulting composite. The effect is a mechanical one complicated by several superimposed effects of a chemical and physical nature. The pure mechanical effect is best understood. It is accounted for in terms of the local strain distortions due to dissimilarities between the matrix and filler material. Various theories have been propounded to explain it, and reviews of these theories are available [1–3]^1^.

In order to test these theories good data are needed for a series of macroscopically homogeneous and isotropic composites containing various known amounts of filler. The filler and matrix materials should be isotropic, and the two elastic constants necessary to characterize each of them should be known. These two elastic constants should preferably be obtained by simultaneous measurements on the same specimen in order to minimize effects due to viscoelasticity and specimen variation.

There are a number of physical and chemical effects that must be considered in choosing an appropriate composite system for testing. The rubbercarbon black system for instance is complicated by the following effects [4–6]: The small carbon particles present in large numbers bond to the rubber molecules and significantly increase the degree of crosslinking of the rubber matrix; the carbon particles associate together in long chains thus producing a structure effect; and the filled rubber composite softens after a previous deformation. For these reasons this system does not provide a satisfactory test of theories dealing with the mechanical effect.

In some cases the presence of the filler particle may induce crystallization, crosslinking or other structural changes in the surrounding shell of polymer matrix, thus introducing an additional phase with consequent changes in mechanical properties [7–11]. This effect should be avoided by using a filler which adheres firmly to the matrix, but does not exercise a long-range influence on the matrix structure. In addition the filler particle dimensions should be large enough that the total filler surface area is comparatively small.

The room temperature behavior of a composite formed at elevated temperatures may be affected by frozen-in stresses due to differences in the thermal expansion properties of the matrix and filler material [12]. In cast composites this effect can be reduced by gelling at room temperature, and cooling slowly after any post cure at elevated temperatures.

The shape and orientation of the filler particles may cause the composition to be anisotropic, hut this effect is easily avoided by using spherical filler particles.

Previous checks of the theories have met some of the above criteria in various ways. For those composites consisting of a rigid filler imbedded in a rubbery matrix of Poisson’s ratio 0.5, the shear modulus of the composite increases as the volume fraction of the filler is increased. This effect is analogous to the increase in viscosity of a suspension of particles in a liquid [13], so that viscosity data such as those provided by Eilers [14] can be used in a partial check of the theories.

Data are also available on composites with a rubbery matrix, but these data are usually incomplete in that only one elastic constant is measured as a function of the volume fraction of filler. In these cases the dependence on filler content of the other constant, that is needed to characterize an isotropic material, cannot be checked. Schwarzl et al. [15] and Waterman [16] however, have provided complete data on a composite system consisting of NaCl crystals imbedded in a rubbery polyurethane matrix, and have compared them with the predictions of van der Poel’s theory [17], Additional data on this material have been provided by Nicholas and Freudenthal [18]. Payne [6] has measured the shear moduli of composites consisting of natural rubber filled with glass spheres, whiting and a “nonstructure” carbon black, and has given a valuable discussion of this system.

When the polymer matrix is in the leathery or glassy state, one can no longer assume as in the rubbery case that Poisson’s ratio of the matrix is close to 0.5. Yet, unless two elastic constants are known for the matrix material, and reasonable values of the constants can be assumed for the filler, theoretical predictions for the composite’s elastic behavior can only be approximate. In much of the data for glassy-matrix composites the constants of the components are not completely specified, and in most cases only one constant characterizing the composite (i.e. Young’s modulus or shear modulus) is measured as a function of filler content. Some useful but usually incomplete data are available [17, 19–22]. More data on glassy-matrix composites are desirable, and for this reason the work reported here was undertaken.

## 2. Materials

The system of composites studied consisted of glass spheres imbedded in and adhering to a glassy epoxy matrix. A series of such materials was fabricated, each member containing a different volume fraction of glass spheres.

The matrix material was formed from the diglycidyl ether of bisphenol-A (DGEBA) hardened with a stoichiometric amount of triethylenetetramine (TETA). The DGEBA resin used (DER-332, Dow Chemical Co.)^2^ was almost pure monomer with an epoxy equivalent weight 172–178. The glass transition temperature of the hardened polymer as determined by dilatometry was 120 °C. The density determined by hydrostatic weighing was 1.187 g/cm^3^ for material stored 185 days at 24 °C and 54 percent R.H.

The filler material consisted of glass spheres with a distribution of diameters in the range 1 to 30 *μ*m. The glass was an optical crown glass, soda lime type, with a silica content not less than 60 percent (Standard Class IV Unispheres No. 4000, Cataphote Corp.). The manufacturer specifies an approximate Young’s modulus for this glass of 7.6 × 10^10^ Pa (11 × 10^6^ lb/in^2^) as measured on a bulk sample. Poisson’s ratio for soda lime glass, given in the literature [23], is 0.23. The density of this glass as determined with a pycnometer is 2.392 g/cm^3^.

Before use the glass spheres were cleaned by passing them near the poles of a powerful permanent magnet a number of times to remove iron impurities. They were washed twice in boiling distilled water and twice in boiling isopropanol, and dried overnight under vacuum at 130 °C. The dried spheres were coated with the coupling agent *γ*-aminopropyltriethoxysilane (A1100, Union Carbide Corp.) as follows: A given weight of spheres was added to an equal weight of freshly prepared 1 percent-solution of A1100 in water. The slurry was stirred for 15 min, filtered, and washed with an equal amount of water. The spheres were then dried overnight under vacuum at 130 °C, and then lightly ground in a mortar to break up agglomerations.

Before use the DGEBA monomer was deaerated in a vacuum oven for at least 1 hour at 60 °C. A weighed amount was transferred to a flask, the glass spheres added, and the slurry stirred under vacuum for an hour to remove air introduced with the spheres. The TETA hardener was added, and the mixture rapidly stirred under vacuum for 5 minutes. The mixture then was carefully poured into a mold formed from clamped glass plates separated by spacers and sealed with rubber tubing. The plates had previously been treated with a mold release agent.

The mold was sealed and the comems allowed to cure for 16 hours at room temperature. Before gelling occurred the mold was rotated at 1 rpm in order to prevent the glass spheres from settling. The material was post cured in an unclamped mold for 24 hours at 75 °C followed by 8 hours at 150 °C. The oven was then turned off and the cured composite allowed to come to room temperature overnight in the oven. According to reported research on similar materials [24, 25] this should produce an almost completely cured resin with only minimal strains introduced by the molding process.

Several series of composites containing volume fractions of glass spheres up to 0.50 were prepared by this process. The content of glass was determined by hydrostatic weighing and found to be uniform throughout each of the cast samples. Test specimens of dumbbell shape [26] with 5-cm gage length, 1.3-cm gage width and 0.5-cm thickness were prepared from the castings, using a high-speed router. Four specimens were obtained from each cast sample.

## 3. Procedure

The specimens were tested on a tensile testing machine at 0.05-cm/min rate of extension and 12.5-cm grip separation. The state of strain in the uniform narrow portion of the specimen was monitored with a strain gage extensometer of nominal 5-cm gage length. The exact value of the initial gage length was determined in situ with a cathetometer. A transverse extensometer employing a small linear variable differential transformer was used to obtain simultaneous measurements of the width of the specimen during test. The amplified outputs of the two extensometers and the load-extension curve of the tensile tester were recorded separately. Simultaneous data values were obtained by putting small simultaneous pip marks on the recorder traces by means of a pushbutton operated pulse circuit.

The data were used to plot stress-strain curves and curves of transverse strain versus longitudinal strain for the specimens. The initial slope of the stress-strain curve was taken as Young’s modulus of the specimen, and the negative of the initial slope of the transverse strain-longitudinal strain curve was taken as Poisson’s ratio. As all of the specimens were subjected to the same low rate of straining during data acquisition, it was believed that the relative effects of viscoelasticity would be small. Therefore viscoelastic effects were not considered in the subsequent analysis.

## 4. Results

During early stages of the research it was assumed that the samples were fully cured, so that tensile properties could be measured 1 or 2 weeks after preparation. The results of these measurements are given in table 1. Each value listed is the average of measurements on the four specimens obtained from each cast sample, and the variation between specimens is expressed as the probable error of a single observation. The probable errors are of the order of 3 percent of the measured value for both Young’s modulus and Poisson’s ratio. The variation from sample to sample however was noticeably greater, even though the extra samples of same filler content were made and measured by the same standardized procedure.

A later series of samples made and measured is listed in table 2. These samples were tested 200 days after preparation to see if there was an aging effect. During this time the samples were stored over a saturated solution of Mg(NO_3_)_2_ · 6H_2_O maintained at the laboratory temperature of 24 °C. This provided an environment of 54 percent relative humidity. As a check on the effect of moisture two extra samples of epoxy matrix material were prepared. One was stored over water, and the other was stored over anhydrous CaSO_4_ in a vacuum desiccator.

Comparison of the values given in tables 1 and 2 shows that there is an age hardening effect. The Young’s moduli tabulated in table 2 are slightly higher than the corresponding moduli in table 1. However the measurement values obtained do not seem to have been affected by the relative humidity of the environment. The Young’s moduli for the three samples stored in different environments are not significantly different.

## 5. Discussion

The data just presented will now be analyzed with the help of a theory due to van der Poel [17], and compared with the predictions of other theories. Let *G* represent the shear modulus, *K* bulk modulus, *E* Young’s modulus, and *ν* Poisson’s ratio of the composite; let the subscripts *f* and *m* refer to the filler and matrix respectively, and let *φ* represent the volume fraction of filler. These elastic constants are interrelated by the equations,

$$E=\frac{9KG}{3K+G}$$ (1)

$$\nu =\frac{3K-2G}{6K+2G}$$ (2)

so that only two constants are required to characterize the initial elastic behavior of an isotropic material. Van der Poel’s theory provides two relationships,

$$K=K(G_{m},K_{f},K_{m},\varphi )$$ (3)

$$G=G(G_{f},G_{m},\nu_{f},\nu_{m},\varphi )$$ (4)

and similar relationships are provided by the other theories.

Relation (3) is also used in the theory of Kerner [27], and is the same as Hashin and Shtrikman’s equation for the highest lower bound of the bulk modulus [28]. However van der Poel’s relation (4) for the shear modulus, as given in the original presentation, was very complicated. A table of values was provided, but this table was limited to materials for which Poisson’s ratio of the matrix was 0.5. In addition there was an error in the derivation. Recently van der Poel’s method has been reexamined, the error corrected, and the method extended for use with matrix materials having any value for Poisson’s ratio [29]. Subsequently the calculation of *G* has been simplified [30].

If the elastic constants *E_m_*, *ν_m_*, *E_f_*, *ν_f_* of the matrix and filler materials are known, the corresponding values *K_m_, G_m_*, *K_f_*, *G_f_* can be calculated by means of relations (1) and (2). *K* and *G* for the composite can be calculated as a function of *φ*, using equations (3) and (4), and the corresponding values of *E* and *ν* then can be calculated from relations (1) and (2). It is these calculated values of *E* and *ν* that are compared with the experimental data.

In order to determine how well the theories agree with the experimental data it is necessary to have accurate values of *E_m_* and *ν_m_* for use in the theoretical calculations. The average of the measured values of *E_m_* and *ν_m_* was not regarded as sufficiently accurate, so more accurate values were obtained by the following extrapolation procedure: The averages of *E_m_* and *ν_m_* were used as a first approximation, and curves of *E* versus *φ* and *ν* versus *φ* were calculated using van der Poel’s theory. These curves were fitted to the experimental data for values of *φ* up to 0.15. This was done by shifting the curves mathematically along the *E* or *ν* axis to obtain a least squares fit with the experimental data. The shape and slope of the curves was maintained constant during the shifting process. The intercepts of the shifted curves provided new values of *E_m_* and *ν_m_*. The process was repeated until convergence was obtained.

The values of *E_m_* and *ν_m_* obtained by this process are: For the data of table 1, *E_m_* = 2.68×10^9^ Pa, *V_m_* = 0.394; for data of table 2, *E_m_* = 3.01 × 10^9^ Pa, *ν_m_* = 0.394. It is apparent from the above values of *E_m_* that age hardening occurred during the time interval between tests at 10 days and 200 days after sample preparation.

**Figure 1:**
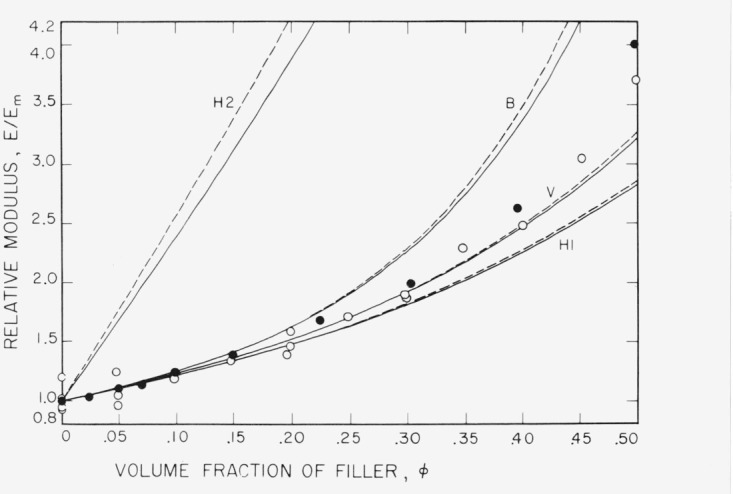
Plot of relative Young’s modulus *E/E_m_* versus volume fraction of filler, from the data of tables 1 and 2. Open circles, data from table 1 using *E_m_* = 2.68 × 10^9^ Pa: solid circles, data from table 2 using *E_m_* = 3.01 × 10^9^ Pa. Dash curves, theoretical values calculated using *E_m_* = 2.68 × 10^9^ Pa; solid curves, theoretical values calculated using *E_m_* = 3.01 × 10^9^ Pa. H1, calculated using Hashin and Shtrikman’s highest lower bounds for shear and bulk moduli; H2 calculated using Hashin and Shtrikman’s least upper bounds for shear and bulk moduli; V, van der Poel’s theory; B, Budiansky’s theory.

Relative moduli *E\E_m_* for the samples are plotted as a function of filler volume fraction in figure 1. Data for the fresh samples (table 1) are plotted as open circles, and for the age hardened samples (table 2) as solid circles. Predicted values from the van der Poel and Budiansky [31] theories and the least upper (curve H2) and highest lower bounds H1 of Hashin and Shtrikman’s theory are shown as pairs of solid and dashed lines. The dashed lines show predicted values for the fresh samples (*E_m_* = 2.68 × 10^9^ Pa), and the solid lines show values for the age hardened samples (*E_m_* = 3.01 × 10^9^ Pa). Predicted values of Kerner’s widely used theory are the same as those of Hashin and Shtrikman’s highest lower bound H1, and have been shown to be approximations to the values calculated using van der Poel’s theory [30].

It is apparent from figure 1 that the values of relative modulus *E/E_m_* calculated using van der Poel’s theory are in good agreement with the measured values for filler volume fractions up to 0.35, and give slightly better predictions than values calculated using the Kerner or Hashin and Shtrikman lower bound theory. At higher filler volume fractions up to 0.50 measured values exceed the van der Poel predictions, but are less than those of Budiansky.

**Figure 2:**
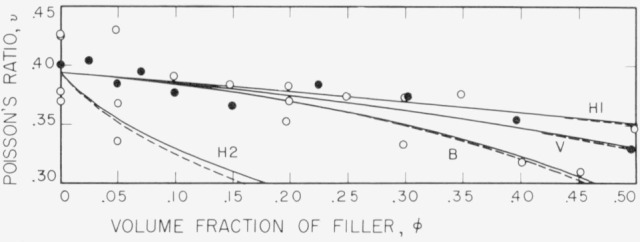
Plot of Poisson’s ratio versus volume fraction of filler, from data of tables 1 and 2. Open circles, data from table 1; solid circles, data from table 2. Dash curves, theoretical values calculated using *E_m_* = 2.68 × 10^9^ Pa: solid curves, theoretical values calculated using *E_m_* = 3.01 × 10^9^ Pa. H1, calculated using Hashin and Shtrikman’s highest lower bounds for shear and bulk moduli; H2, calculated using Hashin and Shtrikman’s least upper bounds for shear and bulk moduli; V, van der Poel’s theory; B, Budiansky’s theory.

Poisson’s ratios are plotted in figure 2. Values for the fresh samples are plotted as open circles, and values for the age hardened samples as solid circles. Predictions of the van der Poel, Budiansky and Hashin and Shtrikman theories are shown as pairs of solid and dashed lines, where the dashed lines depict values for the fresh samples, and the solid lines depict values for the age hardened samples. Predictions of Kerner’s theory are the same as those of the Hashin and Shtrikman curves designated as H1.

The order of the curves in figure 2 is reversed from the order observed in plots of *E/E_m_* versus *φ*. Thus curve H2 calculated using Hashin and Shtrikman’s values for the least upper bounds on *K* and *G* actually becomes a lower bound curve for Poisson’s ratio *ν*, and curve H1 calculated from the highest lower bound values becomes the upper bound curve. Also the curve calculated from Budiansky’s theory lies below that calculated from van der Poel’s theory. The reversal in the order of these curves is a consequence of the decrease in the value of *ν* with increasing *φ*.

Although there is considerable scatter in the data, the curve of values calculated from van der Poel’s theory seems to provide the best fit. These results indicate that the generalized van der Poel theory is better than other current theories in predicting the mechanical effect of filler on the elastic properties of a particulate composite. As the calculations involved in the application of this theory have now been simplified [29, 30], it is hoped that the theory will be more frequently used in the interpretation of experimental data.

## Figures and Tables

**Table 1:** Results for samples tested 1 to 2 weeks after preparation^a^

Volume fraction of filler	Days to test	Young’s modulus	Poisson’s ratio
*φ*		*E* *GPa*	*ν*
0.0000	13	3.21 ± 0.09	0.426 ± 0.008
.0000	7	2.74 ± .11	.425 ± .010
.0000	10	2.53 ±.06	.378 ± .003
.0000	13	2.48 ± .05	.370 ± .009
.0482	7	b3.35 ± .06	b.430 ± .012
.0495	10	2.58 ± .03	.336 ± .014
.0498	13	2.83 ± .03	.368 ± .006
.0985	13	3.19 ± .03	.391 ± .018
.1477	14	3.62 ± .13	.384 ± .010
.1965	7	3.74 ± .04	.353 ± .007
.1983	8	4.25 ± .12	.383 ± .012
.1992	13	3.91 ± .03	.370 ± .008
.2486	14	4.59 ± .10	.374 ± .007
.2980	5	5.09 ± .07	.333 ± .003
.2988	12	5.02 ± .11	b.373 ± .004
.3480	15	6.14± .15	.376 ± .010
.4015	8	6.67 ± .15	.318 ± .012
.4513	12	8.17 ± .23	c.310 ± .005
.4981	14	9.95 ± .90	b.347 ± .010

a Expressed as value ± probable error of a single observation. Each value is the average of measurements on four specimens, except as noted.

b Average of three specimens.

c Average of two specimens.

**Table 2:** Results for samples tested approximately 200 days after preparation^a^, ^b^

Volume fraction of filler	Days to test	Young’s modulus	Poisson’s ratio
*φ*		*E* *GPa*	*ν*
0.0000	198	2.99 ± 0.11	0.401 ± 0.011
.0000	194	c3.00 ± .05	c.403 ± .021
.0000	190	d3.03 ± .05	d.400 ± .005
.0245	203	3.11 ± .02	.404 ± .008
.0494	197	3.32 ± .11	.385 ±.007
.0701	203	3.41 ± .08	.395 ± .006
.0993	207	3.75 ± .09	.377 ± .019
.1494	208	4.19 ± .14	.366 ± .010
.2251	209	5.08 ± .08	.384 ± .020
.3025	209	6.01 ± .21	.375 ± .017
.3960	208	7.94 ± .12	.354 ± .018
.4960	208	12.09 ± .09	.330 ± .013

a Expressed as value ± probable error of a single observation. Each value is the average of measurements on four specimens.

b Specimens were stored at 24 °C and 54 percent R.H. before testing, except as noted.

c Stored in a vacuum desiccator over anhydrous CaSO_4_ at 24 °C.

d Stored over water at 24 °C.

## Footnotes

1 Figures in brackets indicate the literature references at the end of this paper.

2 Certain commercial equipment, instruments or materials are identified in this paper in order to provide an adequate description. In no case does such identification imply recommendation or endorsement by the National Bureau of Standards.
